# Le lymphome primitif de la vessie: un cas clinique

**DOI:** 10.11604/pamj.2014.18.148.4365

**Published:** 2014-06-17

**Authors:** Mouad Statoua, Maha Mokrim, Jihad El Ghanmi, Tariq Karmouni, Khalid El Khadir, Abdellatif Koutani, Ahmed Iben Attya, Hassan Errihani

**Affiliations:** 1 Service d'Urologie B, CHU Ibn Sina Rabat, Maroc; 2 Service d'Oncologie Médicale, Institut National D'Oncologie Chu Ibn Sina, Rabat, Maroc

**Keywords:** Lymphome, vessie, histologie, chimiothérapie, surveillance, Lymphoma, bladder, histology, chimiotherapy, surveillance

## Abstract

Les tumeurs de vessie constituent les tumeurs les plus fréquentes de l'appareil uro-génital chez l'homme, le type histologique est dominé par le carcinome urothéliale, la localisation primitive du lymphome malin est extrêmement rare et constitue souvent une surprise diagnostique quand elle est retrouvée. Nous rapportons à travers cette observation le cas d'un patient pris en charge au sein de notre formation qui a bénéficié, suite a un bilan d'hématurie objectivant une formation bourgeonnante intra vésicale a l’échographie abdominale, d'une résection transurétrale de la vessie d'un polype géant dont les résultats anapathologies sont revenus en faveur d'un lymphome primitif de la vessie, le patient a bénéficié d'une chimiothérapie adjuvante a base de RCHOP, les suites étaient simples et une surveillance cystoscopique et scannographique n'a pas objectivé de récidive avec un recul de 18 mois. Le lymphome primitif de la vessie est une entité rare, sa chimio sensibilité fait de ce type de tumeur une maladie curable, le diagnostic est histologique et vu la rareté de l'affection aucun consensus n'est standardisé.

## Introduction

Les localisations primitives vésicales des lymphomes malins sont extrêmement rares, elles représentent souvent une surprise histologique. Nous rapportons l'observation clinique d'un patient présentant ce type de tumeurs maligne dont la prise en charge après le diagnostic endoscopique a été basée sur une chimiothérapie.

## Patient et observation

**Figure 1:**
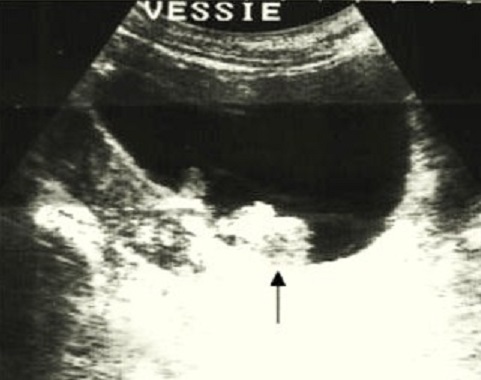
Echographie vésicale montrant le processus intravésical

**Figure 2:**
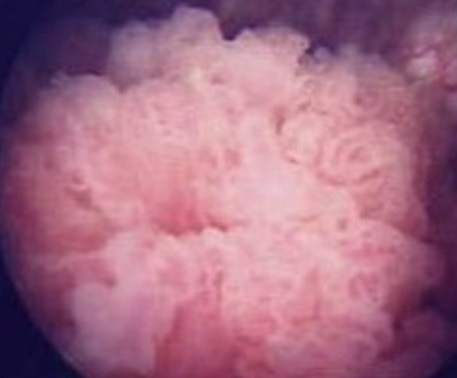
Aspect macroscopique de la tumeur

Notre patient H.R. est âgé de 62 ans, tabagique chronique à raison de 40 PA, bronchiteux chronique, présente une cardiopathie ischémique sous traitement, le patient a présenté des épisodes d'hématuries intermittentes avec brulures mictionnelles en plus de douleurs pelviennes, il a bénéficié d'une échographie réno-vésico-prostatique (Figure 1) qui a objectivé la présence d'une masse d’écho structure tissulaire avec un petit rein droit dédifférencié, un bilan de crase a été réalisé qui est revenu normal, la fonction rénale été perturbée avec un taux d'urée à 0,57g/l et une créatininémie à 23,2 mg/l. Le patient a été hospitalisé dans le service d'urologie pour prise en charge de sa tumeur de vessie où un geste de résection trans-urétrale de la tumeur de vessie a été réalisé, au cours de la cystoscopie on a diagnostiqué un processus intravésicale papillaire occupant la face latérale gauche de la vessie faisant presque 5 cm (Figure 2) et une localisation au niveau du dôme vésicale, un geste de résection complète a été réalisé. La fonction rénale s'est nettement améliorée après le geste de résection et réhydratation. Le résultats d'anatomopathologie des copeaux de résection sont revenu en faveur d'un lymphome malin a grandes cellules B avec a l'immunomarquage les anticorps anti CD 20 et les anti CD 5 positif les anti Ki67 positive de plus de 60% et les anti CD10 et les anti cycline D1 négatif. Un bilan d'extension a été réalisé pour rechercher une localisation secondaire notamment une BOM qui est revenu négatif et une TDM thoraco-abdomino-pelvienne qui a objectivé la présence d'un processus lésionnel de la paroi antérieure et latérale gauche de la vessie sans extension au plancher vésical sans ADP ni pelvienne ni lombo-aortique ni thoracique et qui a mis en évidence la présence d'un anévrysme de l'aorte abdominale sous rénale mesurant 70X71X111 cm qui n'a pas été opéré vu la FEVG du malade très basse a 30%. Notre patient a été adressé au service d'oncologie médicale pour complément de prise en charge où il a reçu 4 cures RCHOP et on a constaté une bonne réponse clinique avec disparition de l'hématurie, radiologique sur les contrôles echographiques et scannographiques qui ont objectivé la disparition complète de la lésion vésicale ainsi que cystoscopique avec un recul de 18 mois.

## Discussion

Les lymphomes primitifs de la vessie dont le premier cas a été rapporté en 1885 [1, 2] sont exceptionnels. Cette faible prévalence serait du à la faiblesse de cet organe en tissus lymphoïdes [3, 4]. Ils sont apparentés aux lymphomes du MALT (Mucosa Associated Lymphoid Tissue), bien qu'aucun antigène n′a été encore individualisé pour la vessie [3, 5]. Plusieurs hypothèses physiopathologiques ont été lancé pour pouvoir expliquer la pathogénie de ce type de lymphome mais devant la rareté de cette localisation on n'a pas encore chiffré la physiopathologie ainsi certains auteurs rapporte la prolifération lymphoïdes à ce niveau aux cystites a répétition mais cet antécédent n'est retrouvé que chez 20% des patients atteints de lymphome vésicale [6]. De plus des études histologiques ont montré une prolifération tumorale manifeste au niveau des couches musculaires profondes et non pas en sous muqueux [7]. La possibilité d'un reliquat embryonnaire cloacale a été aussi évoquée [3]. Les lymphomes primitifs de la vessie surviennent surtout chez les femmes après 40 ans les hommes sont 6 fois moins touchés [3, 7], cliniquement l'hématurie est souvent le signe révélateur (80% des cas), d'autres signes irritatifs peuvent être présent, la douleur et l'altération de l’état général sont rare. Le stade d'insuffisance rénale reste exceptionnel. Sur le plan biologique les LDH demeurent normal même à un stade localement avancé [1, 8]. Sur le plan morphologique l’échographie vésicale couplé à l'examen cystoscopique reste les examens de première intention mais il n'y a aucune particularité structurale qui oriente vers les lymphomes [1, 4], la certitude diagnostique est portée par l'examen histologique des copeaux de résection[8].

La majorité des lymphomes primitifs vésicaux sont non hodgkiniens [3]. Quelques rares cas de maladie de Hodgkin localisée à la vessie ont été décrits [3, 9]. Les types histologiques les plus fréquents sont les lymphomes diffus à grandes cellules ou les lymphomes à petites cellules, qui représentent chacun 30% des cas. Les lymphomes à différenciation plasmocytaire ou folliculaires sont retrouvés respectivement dans 20% et moins de 10% des cas [10]. Il s'agit le plus souvent de lymphomes malins non hodgkiniens de phénotype B, comme dans les autres lymphomes extra-nodaux non cutanés [3, 10]. Les lymphomes sont pour la plupart chimiosensible et donc la chimiothérapie reste le traitement de première intention envisagé [11], le protocole le plus utilisé est rituximab-CHOP (protocole utilisé chez notre patient), en cas de récidive locorégionale on peut prescrire une deuxième chimiothérapie plus lourde ou une radiothérapie seule ou complétant une chirurgie d'exérèse puisque la radiothérapie ne constitue pas le traitement de référence. La place de la chirurgie est limitée: soit à visée palliative, en particulier en cas d'obstruction [12], soit pour certaines équipes en cas de tumeurs localisées et de bas grade, une résection trans-uréthrale suivie d′une radiothérapie externe a pu être proposée. Par contre, pour les tumeurs étendues ou de haut grade la chimiothérapie seule est recommandée [12]. Le pronostic dépend essentiellement du type histologique et du volume tumoral au moment du diagnostic, les lymphomes primitifs de la vessie, tous types histologiques confondus, sont associés a un taux de survie compris entre 68 et 73% à 1 ans, et compris entre 30 et 64% à 5 ans [13]. Cependant, la variété des protocoles thérapeutiques proposés et la rareté de cette pathologie avec des études de faibles effectifs ne permettent pas aujourd'hui de connaître les caractères évolutifs du lymphome vésical avec précision.

## Conclusion

Le lymphome primitif de la vessie est une forme rare des tumeurs de la vessie, son diagnostic repose sur une preuve histologique vu qu'il n'existe aucun signe clinique, radiologique ou endoscopique spécifique. La chimiothérapie reste le traitement de première intention et le pronostic est souvent favorable sur la tumeur primitive vu sa chimio et radiosensibilité.

## References

[1] Peyromaure M, Van Glabeke E, Leblond V, Barrou B, Delcourt A, Richard F (2000). Le lymphome primitif de la vessie. Progrès en Urologie.

[2] Eve FS (1885). Two cases of sarcoma of the bladder. Trans Path Soc Lond..

[3] Bitker O, Bagnis C (1992). Lymphome primitif vésical à propos d'un cas. Prog Urol..

[4] Brice P, de Kerviler E (2007). Lymphomes de l'appareil urogénital. Ann Urol..

[5] Kempt On CL, Kurtin PJ, Inw Ards DJ, Wollan P, Bostwick DG (1997). Malignant lymphoma of the bladder: evidence from 36 cases that low-grade lymphoma of the MALT -type is the most common primary bladder lymphoma. Am J Surg Pathol..

[6] Simpson RH, Bridger JE, Anthony PP, James KA, Jury I (1990). Malignant lymphoma of the lower urinary tract: A clinicopathological study with review of the literature. Br J Urol..

[7] Heaney JA, De Lellis RA, Rudders RA (1985). Non-Hodgkin lymphoma arising in lower urinary tract. Urology.

[8] Lopez-guillermo A, Colomo L, Jimenez M, Bosch F, Villamor N, Arenillas L (2005). Diffuse large B-cell lymphoma: clinical and biological characterization and outcome according to the nodal or extranodal promary origin. J Clin Oncol..

[9] Sosna J, Lossos IS, Libson E (2000). Hodgkin's lymphoma of the urinary bladder. Clin Radiol..

[10] Ohsaw AM, Aozasa K, Horiuchi K, Kanamaru A (1993). Malignant lymphoma of bladder: report of three cases and review of the literature. Cancer.

[11] Wohrer S, Dirch J, Hejna M (2003). Traitement du lymphome de MALT avec le mitoxantrone, le chlorambucil et la prédnislone. Ann Oncol..

[12] Coiffier B, Lepage E, Brière J, Herbrecht R, Tilly H, Bouabdallah R (2002). CHOP chemotherapy plus rituximab compared with CHOP alone in elderly patients with diffuse large-B-cell lymphoma. N Engl J Med..

[13] Guthman DA, Malek RS, Chapman WR, Farrow GM (1990). Primary malignant lymphoma of the bladder. J Urol..

